# Genome-Wide Ultrabithorax Binding Analysis Reveals Highly Targeted Genomic Loci at Developmental Regulators and a Potential Connection to Polycomb-Mediated Regulation

**DOI:** 10.1371/journal.pone.0161997

**Published:** 2016-08-30

**Authors:** Daria Shlyueva, Antonio C. A. Meireles-Filho, Michaela Pagani, Alexander Stark

**Affiliations:** Research Institute of Molecular Pathology (IMP), Vienna Biocenter (VBC), Vienna, Austria; Instituto Gulbenkian de Ciencia, PORTUGAL

## Abstract

Hox homeodomain transcription factors are key regulators of animal development. They specify the identity of segments along the anterior-posterior body axis in metazoans by controlling the expression of diverse downstream targets, including transcription factors and signaling pathway components. The *Drosophila melanogaster* Hox factor Ultrabithorax (Ubx) directs the development of thoracic and abdominal segments and appendages, and loss of Ubx function can lead for example to the transformation of third thoracic segment appendages (e.g. halters) into second thoracic segment appendages (e.g. wings), resulting in a characteristic four-wing phenotype. Here we present a *Drosophila melanogaster* strain with a V5-epitope tagged Ubx allele, which we employed to obtain a high quality genome-wide map of Ubx binding sites using ChIP-seq. We confirm the sensitivity of the V5 ChIP-seq by recovering 7/8 of well-studied Ubx-dependent *cis*-regulatory regions. Moreover, we show that Ubx binding is predictive of enhancer activity as suggested by comparison with a genome-scale resource of *in vivo* tested enhancer candidates. We observed densely clustered Ubx binding sites at 12 extended genomic loci that included ANTP-C, BX-C, Polycomb complex genes, and other regulators and the clustered binding sites were frequently active enhancers. Furthermore, Ubx binding was detected at known Polycomb response elements (PREs) and was associated with significant enrichments of Pc and Pho ChIP signals in contrast to binding sites of other developmental TFs. Together, our results show that Ubx targets developmental regulators via strongly clustered binding sites and allow us to hypothesize that regulation by Ubx might involve Polycomb group proteins to maintain specific regulatory states in cooperative or mutually exclusive fashion, an attractive model that combines two groups of proteins with prominent gene regulatory roles during animal development.

## Introduction

One of the most fascinating aspects of developmental gene regulation is the specification of animal body segment identity by homeobox domain containing transcription factors (TFs), including homeotic Hox factors. In many animals, Hox factors are arranged linearly in one or more genomic clusters and their sequential order along the genome sequence typically reflects their expression domain along the animals’ anterior-posterior axes. Their role in specifying segment identity has been revealed genetically by mutations in Hox factors that lead to homeotic transformations [1,2]. For example, in *Drosophila melanogaster*, dominant mutations in the Antennapedia (Antp) locus lead to transformations of antennae to legs [3], recessive loss-of-function mutations in Antp transform the second leg into antenna [4], and Ultrabithorax (Ubx) mutations transform the balancing organs halteres into a second pair of wings [5,6].

Such prominent phenotypes made the study of Hox factors and their regulatory targets important and attractive. Genetics established that Hox factors exhibit “posterior prevalence”, a regulatory hierarchy in which more posterior Hox genes repress more anterior ones and are dominant in specifying segment identity [2,7]. A few direct targets and their regulatory elements have been identified and studied in detail [2,8,9] and microarray analyses after ubiquitous overexpression or misexpression of Hox factors have revealed putative regulatory targets genome-wide [10,11]. Since extensive cross-regulation complicated the interpretation of gain- and loss-of-function studies, the binding site locations of Ubx and Dfd have been determined in *Drosophila* embryos and dissected imaginal discs by chromatin immunoprecipitation followed by microarray hybridization (ChIP-chip) [12–14] or by next-generation sequencing (ChIP-seq) [15]. These approaches were either based on antibodies against Ubx and Dfd [13–15] or made use of a protein trap line that contained a YFP insertion in the endogenous Ubx locus [12]. In this line, YFP appeared to recapitulate Ubx expression and flies homozygous or hemizygous for the Ubx-YFP allele were reported to exhibit reduced viability but only weak morphological phenotypes, which suggested that Ubx function was substantially normal [12].

The studies focused on the binding of Ubx in different tissues and/or analyzed the DNA sequence motifs, putative partner TFs, and chromatin features that are involved in the targeting of Ubx or Dfd to their binding sites [12,13,15]. The authors reported Ubx target gene networks, which for example confirmed that Ubx appeared to regulate several signaling pathways and dissected the *cis*-regulatory motif requirements and partner TFs involved in Ubx and Dfd binding and enhancer function.

Here we determine the location of Ubx binding sites in the entire genome of *D*. *melanogaster* embryos using ChIP-seq with antibodies against the heterologous V5 peptide and a *Drosophila melanogaster* strain in which we V5-epitope tagged the endogenous *Ultrabithorax* (*Ubx*) gene using homologous recombination. This revealed specific binding sites with high signal-to-noise ratios, which recovered 7 out of 8 known Ubx-dependent enhancers and were highly predictive of *in vivo* enhancer activity. Given the quality of the individual Ubx binding sites, we analyzed their genomic locations in detail and found that the established regulation of other Hox genes by Ubx is direct and mediated via many individual Ubx binding sites. Ubx also binds in close proximity of many Polycomb complex genes and to known Polycomb response elements (PREs) and Ubx binding sites show significant enrichment of Polycomb and Pleiohomeotic binding genome-wide, which we speculate could reflect a role of Hox genes in directing or antagonizing Polycomb-mediated developmental gene silencing.

## Results

### Tagging of the endogenous Ubx locus by homologous recombination

**Fig 1:**
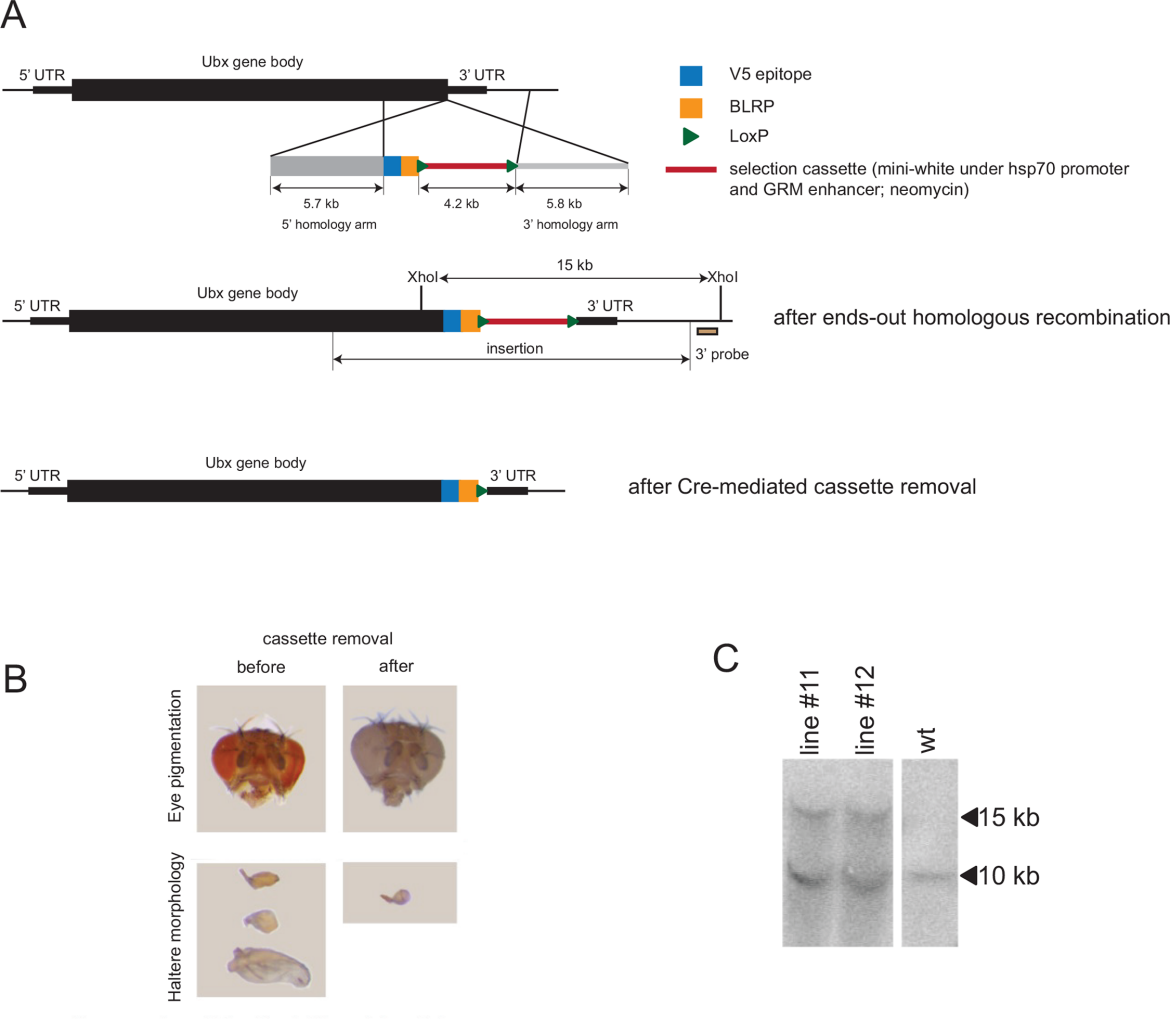
Creation of an epitope-tagged Ubx allele by homologous recombination. (A) Design of the targeting construct (top), the integration to the endogenous Ubx locus and the location of the probe for Southern blotting (middle) and the locus after the cassette removal (bottom). (B) The eye color and haltere morphology for candidate flies before (left) and after cassette removal (white-eyed fly) (right). (C) Southern blot confirming the correct integration for two independently recombined *Drosophila* lines #11 and #12 (heterozygous for the insert). w^1118^ flies were used as a control.

To study Ubx binding throughout *Drosophila* embryogenesis, we first established a *Drosophila melanogaster* strain in which we tagged the endogenous *Ultrabithorax* (*Ubx*) gene with a V5 peptide using homologous recombination (Fig 1A). This strain, which is homozygous for the tagged Ubx allele (see below), should allow for ChIP with high sensitivity and specificity, independently of antibodies against the Ubx protein itself and without altering endogenous Ubx function. We chose to target the C-terminus that is shared between all known transcript isoforms and appears to allow the addition of peptide tags without impacting Ubx function [16].

We first inserted the peptide tag and a selection cassette that was flanked by loxP sites using end-out homologous recombination [17–19] (Fig 1A and Materials and Methods). We selected flies that contained the targeting construct based on eye-color, which varied from dark red to orange, which might indicate various degrees of transcriptional repression of the selection marker in the flies’ eyes (Fig 1B). Interestingly, flies with orange eyes also had changes the morphology of their halteres, including increases in size and transformations to wings (Fig 1B), i.e. homeotic transformation characteristic for *Ubx* loss-of-function alleles [5,6]. This suggested that the cassette was integrated correctly into the Ubx locus, which we confirmed by Southern blot analysis (Fig 1C). Importantly, the haltere phenotype was reversed when we removed the selection cassette (Fig 1B) and flies heterozygous or homozygous for the tagged allele both had wildtype haltere morphology, suggesting that the peptide-tag–in contrast to the entire selection cassette–does not interfere with Ubx function. Taken together, we successfully tagged the 3’ end of *Ubx* and the tagged TF was functional as indicated by the wildtype phenotype in homozygous knock-in flies.

### Characterization of genome-wide Ubx binding in Drosophila embryos

To determine Ubx binding sites genome-wide, we collected embryos of the homozygous tagged strain (0–16 hours post fertilization [hpf]) and performed ChIP-seq with an anti-V5 antibody. Two replicate ChIP-seq experiments from independent embryo collections showed strong and specific enrichments (peaks) and were highly similar with a Pearson correlation coefficient [PCC] 0.86 between the genome-wide read coverage of the two replicates, demonstrating the reproducibility of the approach. We merged both replicates and identified genomic regions that were significantly enriched for Ubx binding (‘peaks’) with peakzilla [20]. We obtained 5282 peaks (peakzilla score ≥3), of which 1479 peaks were particularly strong with a score ≥5. To control for antibody-specificity and to obtain an estimate of the respective false-discovery rates for both score thresholds, we also performed the experiments with embryos from a non-tagged *D*. *melanogaster* strain (denoted hereafter as mock). This yielded 5 peaks with a score ≥3 and no peak with a score ≥5, demonstrating the specificity of the anti-V5 antibody and our approach and suggesting that the false discovery rates (FDRs) for peaks identified with the two score thresholds were 0.10% and 0.07%, respectively.

**Fig 2:**
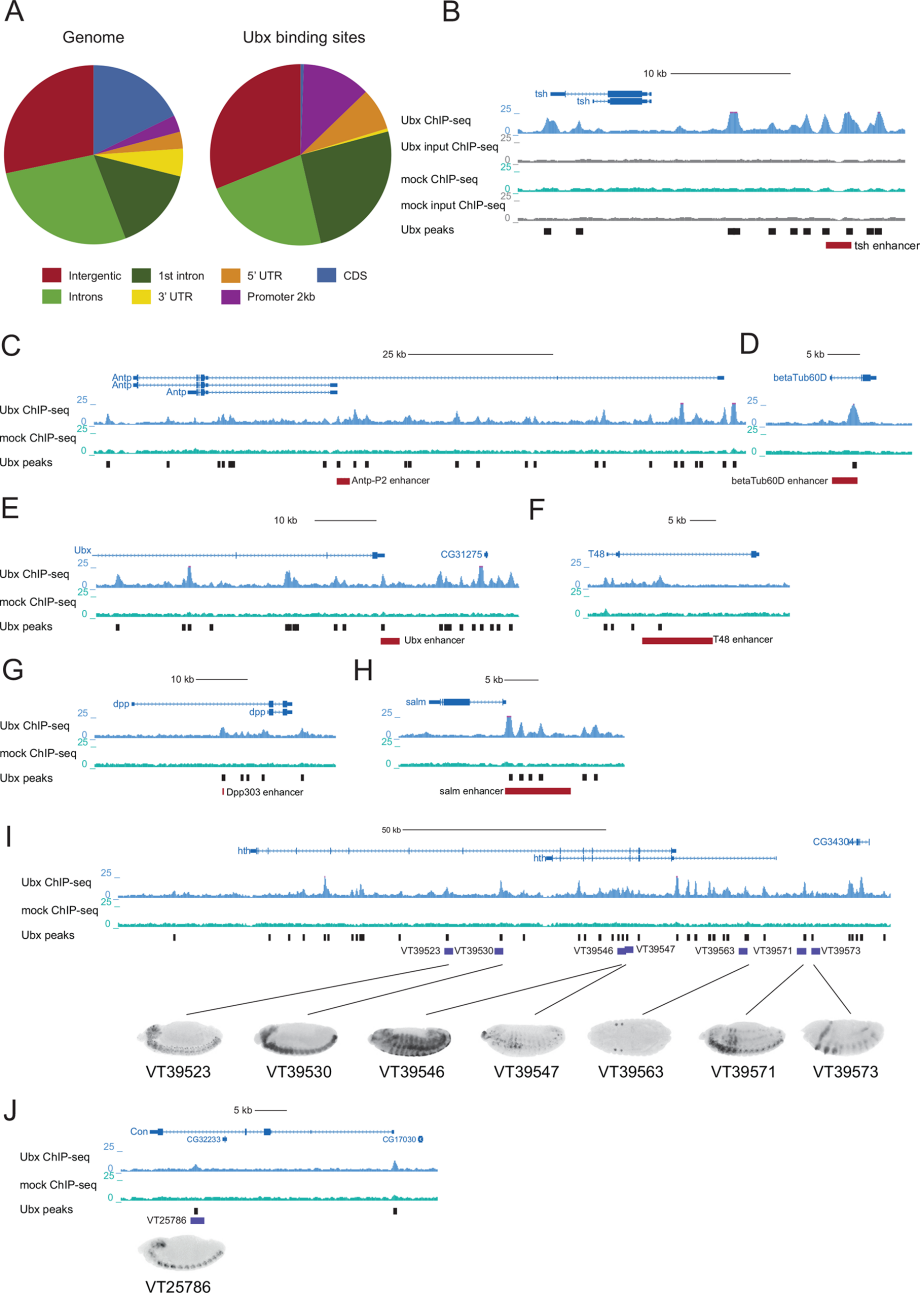
Genomic location of Ubx binding sites and recovery of known Ubx-dependent enhancers. (A) Genomic distribution of Ubx peaks (right) in comparison to the genome (left). (B-H) UCSC Genome Browser screenshots [83] of Ubx (blue), mock (green) ChIP-seq fragment density tracks and the Ubx peak calls at known Ubx-dependent enhancers (red bars) (see the main text for references). Panel (B) also contains the fragment density tracks for the two input samples (grey). (I) UCSC Genome Browser view of the *hth* locus and examples of Ubx-bound embryonic enhancers and their activity patterns [22]. (J) UCSC Genome Browser view of the *Con* locus and Ubx-bound embryonic enhancer [22]. Vienna tiles (VT) [22] are marked by slate blue boxes and their ID numbers are indicated.

The Ubx binding sites were predominantly located in introns (41.8%) or intergenic regions (30.9%) and substantially depleted in coding regions and 3’UTRs, as expected for transcription factor binding sites and transcriptional enhancers [21–24] (Fig 2A). Importantly, the analysis recovered 7 out of 8 known Ubx-dependent *cis*-regulatory regions and binding sites near genes that loss- and gain-of-function studies suggested to be regulated by Ubx [10]. Moreover, *de novo* motif analysis of the Ubx-bound regions (score ≥5) revealed a motif resembling the previously identified canonical Hox motifs (S1 Fig). Among other motifs we observed GAGA and CA-rich motifs that were previously reported to be associated with enhancers in general [25,26].

The identified Ubx binding sites corroborate and provide putative molecular explanations for several long-standing observations, for example within Hox gene loci. Ubx binding to the promoter proximal part of *Antp*-P2 (Fig 2C) suggests that the proposed negative regulation by BX-C genes [27] could indeed be direct and mediated at least in part by Ubx. Similarly, binding of Ubx to its own promoter (Fig 2E) suggests that Ubx directly regulates its own expression, consistent with previous evidence that the *Ubx* promoter is involved in regulation of *Ubx* expression in the visceral mesoderm [28] and that this sequence can be bound by homeodomain-containing proteins [28,29].

The first published chromatin immunopurification with anti-Ubx antibody revealed two transcripts directly regulated by Ubx: *Transcript 48* (*T48*) and 35 (or *Connectin*, *Con*) [30]. We confirmed that Ubx binds to the *T48* enhancer not only *in vitro* [31] but also *in vivo* (Fig 2F). In contrast, we did not observe binding to a putative *Con* enhancer [32], and the respective DNA sequence indeed did not show any enhancer activity during embryogenesis [22]. Instead, we detected a Ubx binding site in a *Con* intron and the corresponding sequence was active in the embryonic ventral nervous cord and in brain lobes, recapitulating *Con* expression pattern in the nervous system [32] (Fig 2J). Similarly, while we did not detect binding at a putative *Dll* enhancer reported to be repressed by BX-C genes in abdominal segments [33], we observed Ubx binding sites more proximally to the *Dll* transcription starting site.

An intronic enhancer of *beta-tub60D* [34] was also bound by Ubx (Fig 2D), confirming the direct mode of regulation proposed previously based on *Ubx* gain- and loss-of-function experiments [34]. We also detected Ubx at well-characterized *tsh* (Fig 2A) and *dpp* enhancers (Fig 2G), which had been suggested to be positively regulated by Ubx based on DNaseI protection assays and enhancer assays of the wildtype enhancers and mutant variants [35,36].

In addition to the small number of regulatory regions proposed to be under direct control of Ubx, hundreds of transcripts have been reported to respond to Ubx misexpression [10]. For example, *hth* was shown to be under negative control of Ubx and abd-A [37] and we indeed detected a large number of Ubx peaks in *hth* locus, many of which (17 out of 26) were active enhancers during embryogenesis with activity patterns reminiscent of *hth* expression [22,38] (Fig 2I).

Finally, several Ubx binding sites in a 10 kb embryonic enhancer upstream of *spalt major (salm)* [39] suggests that Ubx might regulate *salm* not only in haltere imaginal discs [40] but potentially already at embryonic stages (Fig 2H).

Taken together, we obtained high quality Ubx ChIP-seq data that confirmed previous observations regarding Hox-dependent gene regulation and provided further molecular insights into direct binding and regulation by Ubx.

### Ubx binds predominantly to active enhancers and Ubx binding is predictive of enhancer activity

The binding of TFs detected by ChIP-based methods does not imply functionality and not all TF-bound regions correspond to active enhancers [41,42]. To test which proportion of Ubx binding sites coincide with active *cis*-regulatory elements, we used genome-scale resource of 7705 DNA fragments (Vienna tiles or VTs) tested in a reporter assay and imaged throughout *Drosophila* embryogenesis [22].

**Fig 3:**
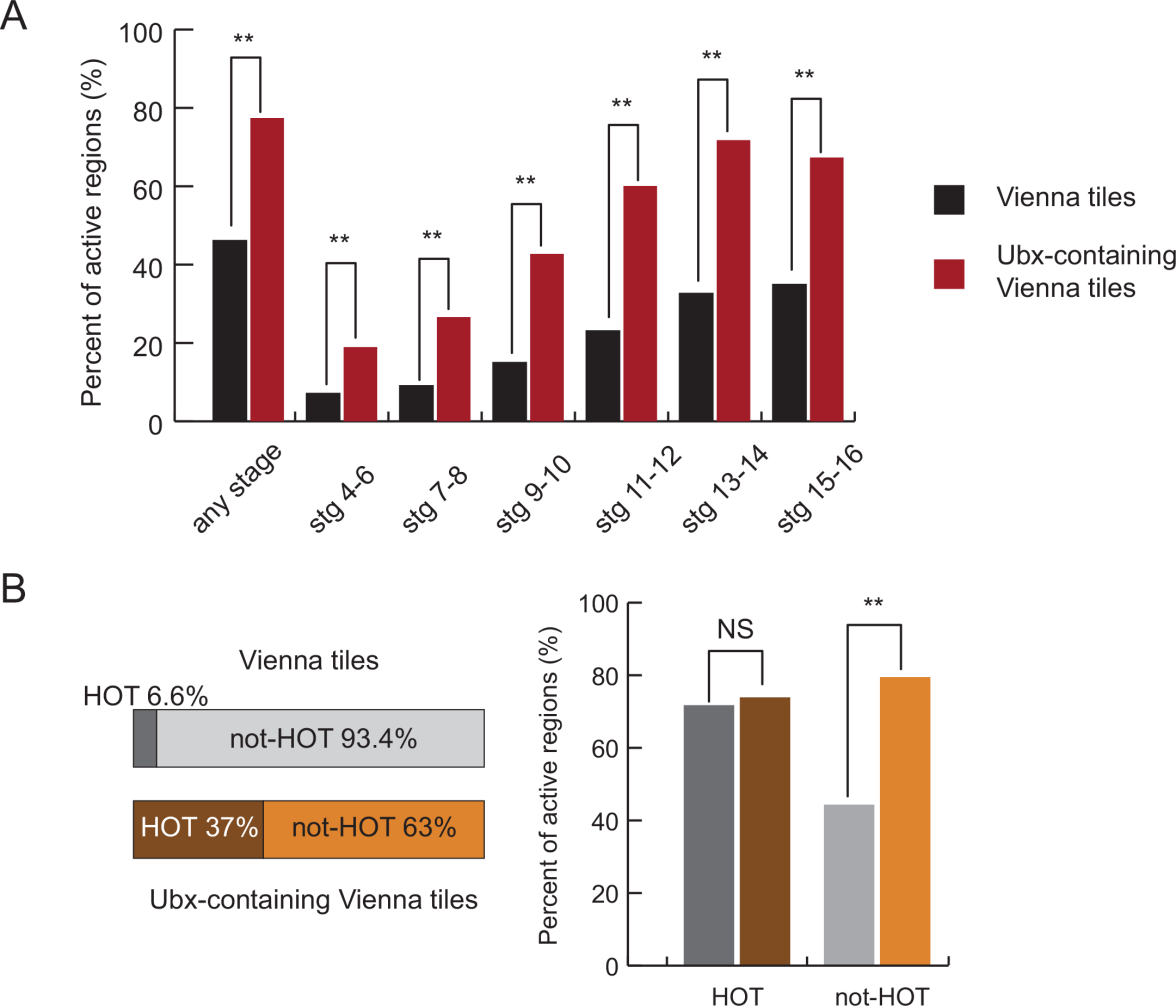
Ubx binds to active enhancers. (A) The bar plot shows the percentage of Vienna tiles (VTs) without or with Ubx binding sites (black and red bars, respectively) that is active at any stage of embryogenesis (left) or at the indicated embryonic stages. Hypergeometric p-value: **P<10^−10^. (B) The left panel shows the fraction of all VTs (top) and the fraction of Ubx-bound VTs (bottom) that overlaps HOT regions (dark shading). The bar plot on the right shows the percentage of active tiles for the four subsets of VTs defined on the left panel. NS–not significant. Hypergeometric p-value: **P<10^−19^.

Of all 248 VTs that overlapped at least one Ubx summit (score ≥5), 192 (77.4%) were active, a 1.7-fold increase (hypergeometric p = 1.7x10^-24^) compared to all VTs of which 46.2% were active (3557 out of 7705) [22] (Fig 3A). The increase was even more prominent when examining the fraction of active VTs at each of the developmental stage intervals separately, which was on average 2.5 times higher for Ubx-bound VTs (Fig 3A). We observed a similar proportion of active VTs when Ubx peaks with score ≥3 were used (75.3%, 552 out of 733, which is again highly significantly different from all VTs [hypergeometric p = 7.8x10^-64^] but not from the ones with score ≥5 above [hypergeometric p = 0.20]).

It was recently shown that TF binding detected by ChIP had a tendency to accumulate at specific genomic regions, termed HOT regions (highly occupied targets) [14,41]. Interestingly, such regions were shown to function as transcriptional enhancers in *Drosophila*, but the functional contribution of each bound TF remained unclear as the HOT regions’ activity patterns did not always coincide or were consistent with the bound TFs’ expression patterns [41]. Our observation that Ubx binding was predictive of enhancer activity was also true when we analyzed the 63% VTs that contained Ubx binding sites but no HOT regions separately (Fig 3B): 79% of Ubx-bound VTs that did not contain any HOT region were active compared to only 44% of all such VTs. The difference was much less pronounced for Ubx-bound VTs that also contained HOT regions (Fig 3B), as HOT regions are frequently active more generally [41].

### Multiple Ubx binding sites in Hox gene loci

**Fig 4:**
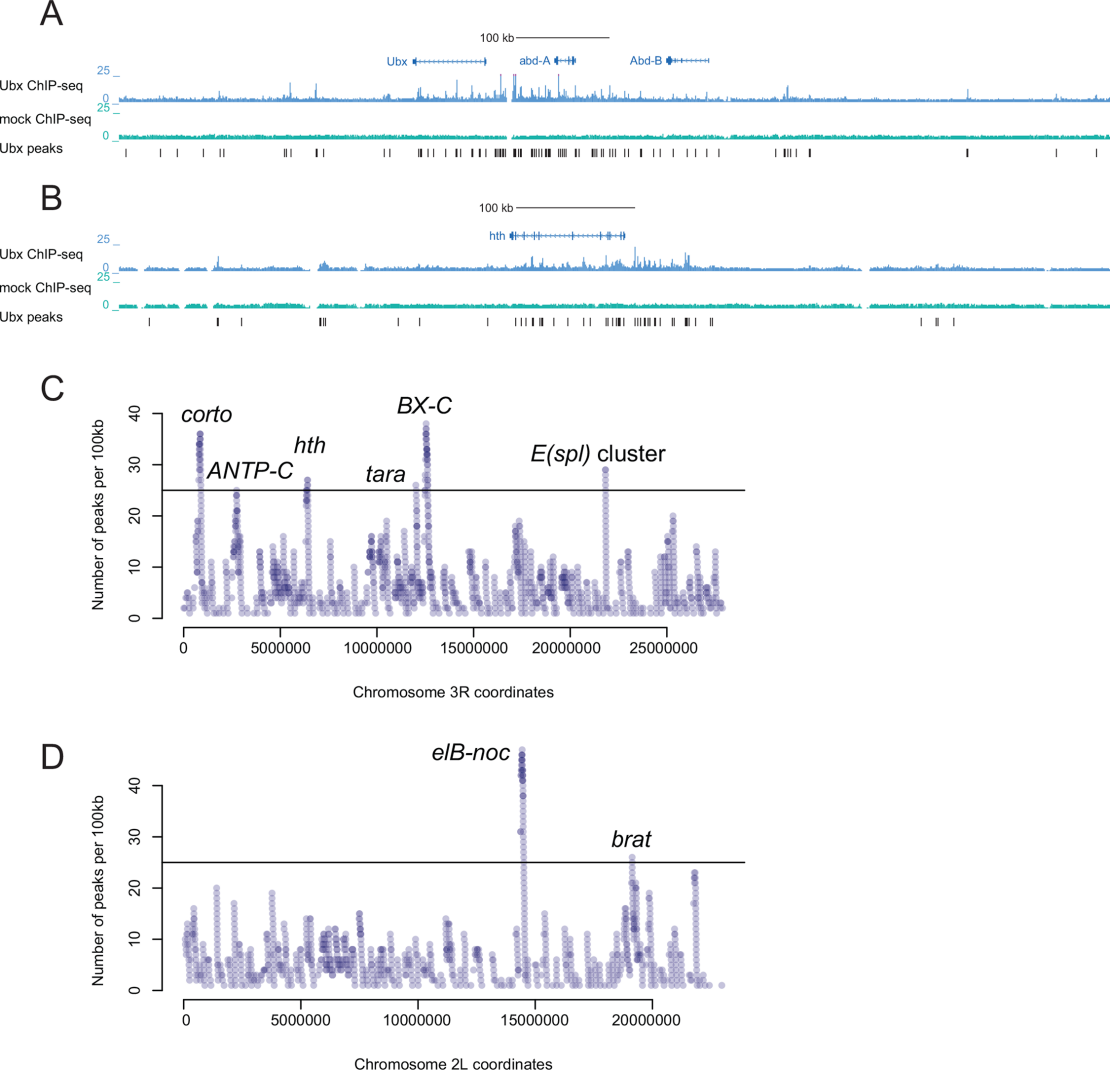
Clustered Ubx binding sites at the genomic loci of important developmental regulators. (A, B) UCSC Genome Browser screenshots at BX-C and *hth* gene loci show ChIP-seq fragment density tracks and Ubx peak calls, revealing many clustered binding sites. (C, D) The number of Ubx peaks per 100 kb on chromosomes 3R and 2L (each 100 kb window starts at a Ubx peak, covering all possible windows that contain at least one Ubx peak). The plots for chromosomes 2R, 3L, X and 4 are in S1 Fig Representative genes for all windows containing ≥25 Ubx peaks are labeled.

One of the prominent features of Hox factors is their extensive cross-regulation [7,8,43]. For example, Ubx was shown to regulate its own transcription [28] and that of *Antp* [27]. We therefore first analyzed Ubx binding sites within the ANTP-C and BX-C loci. Interestingly, the 350kb ANTP-C between *lab* and *Antp* contained 50 Ubx binding sites with scores ≥3, of which 22 had high enrichment scores ≥5, a substantial enrichment compared to the 3 binding sites we observed per 100kb window on average (4.8-fold; Poisson P-value P = 1.8x10^-18^). Furthermore, the 340kb BX-C locus between *Ubx* and *abd-B* contained 73 binding sites, 43 of which were strong (Fig 4A). Importantly, only 30% and 19% of the Ubx binding sites in ANTP-C and BX-C, respectively, coincided with highly occupied target (HOT) regions [14,41], suggesting that the observed enrichment was specific to Ubx and neither due to cross-linking artifacts [44–46] nor shared by many other TFs.

### Clustered Ubx binding sites at highly targeted genomic loci (HTGLs) around developmental regulators and genes related to the Polycomb complex (Pc)

To systematically determine genomic regions that contain clustered Ubx binding sites, we counted the number of peaks in 100 kb windows genome-wide (Fig 4C and 4D, S1 Fig). This revealed two non-overlapping 100 kb windows with 25 Ubx binding sites (score ≥3) or more on chromosome 2L, one on chromosome 2R, and three and six on chromosomes 3L and 3R, respectively (Fig 4C and 4D, S1 Fig).

The two windows with prominent Ubx binding site clusters on chromosome 2L overlapped the gene loci of *elB-noc* and *brat* (Fig 4D). *elB* and *noc* were suggested to play a role in cell proliferation [47] and necessary for the appendage formation [48]. *Brat* is known to regulate post-transcriptional gene expression [49,50] and its mutations caused defects in abdominal segments [50]. The Ubx binding site cluster on chromosome 2R spanned the *sbb* and *tango8* locus, and the clusters on 3L are near the apoptotic genes *scyl* and *chrb* and *W*, *grim* and *rpr*. *Scyl* and *chrb* were previously shown to be de-repressed in *Ubx*, *abd-A* and *Abd-B* mutant flies [51] (S1 Fig). Apoptosis is necessary for the maintenance of segments boundaries, and Ubx–similarly to Dfd and Abd-B [52]–might be linked to its regulation. The third Ubx-rich cluster on chromosome 3L contained *tonalli* (*tna*), a Trithorax group gene that was identified together with *taranis (tara)* (see below) and mutations of which induced homeotic transformations [53]. Besides clustered Ubx binding sites in BX-C, chromosome 3R contained multiple Ubx binding at the *hth* locus (Fig 4B). Hth is a known partner of Hox factors, which has been reported to modulate the specificity of Hox factor binding *in vivo* [9], and our data suggest that Ubx might directly regulate *hth* via a large number of binding sites. Another noticeable cluster on 3R is in the *Enhancer of split (E(spl))* complex, which is a genomic cluster of basic helix-loop-helix (bHLH) transcription factors that are involved in Notch signaling and which are regulated by Ubx in haltere [12,54] (Fig 4C).

**Fig 5:**
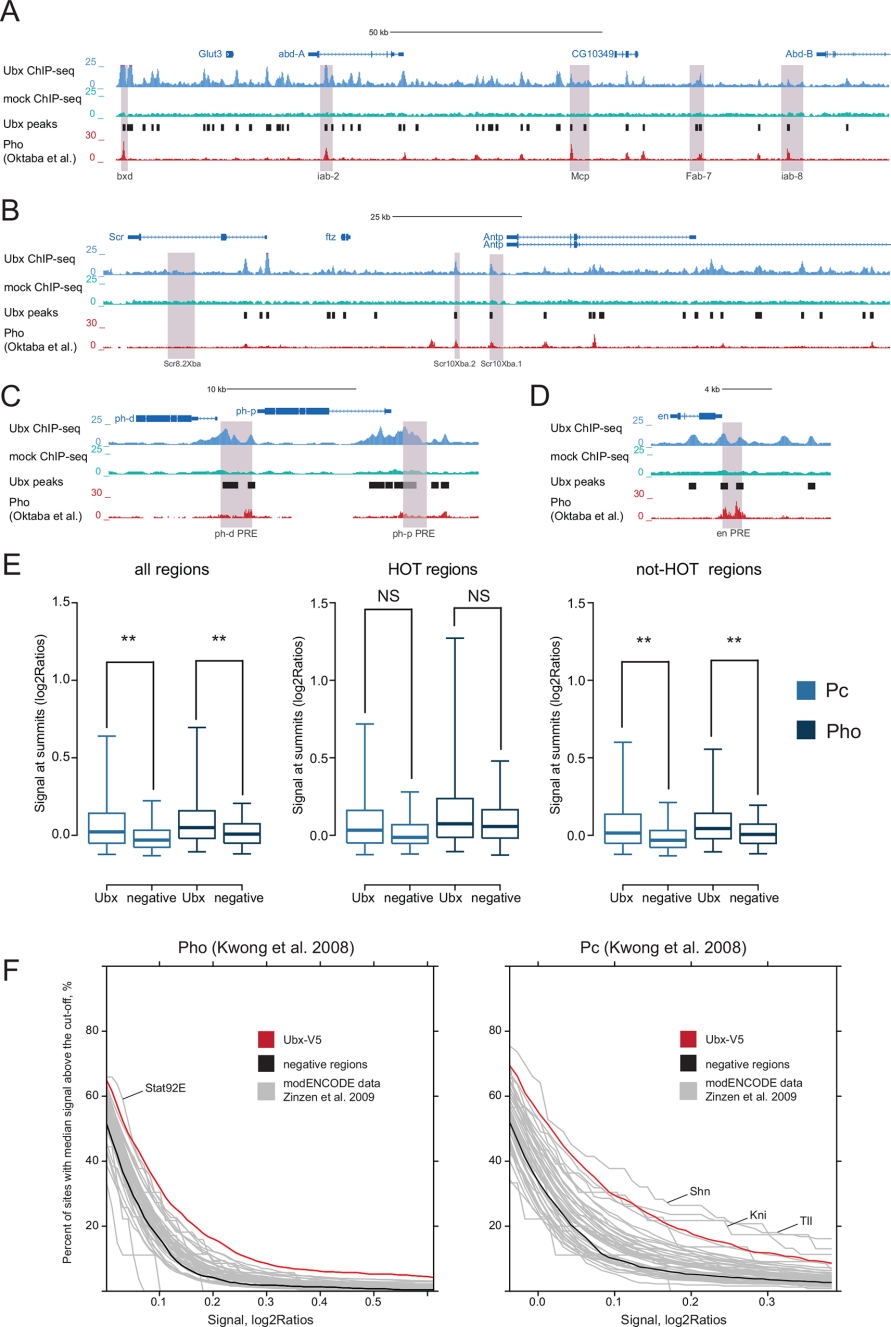
Strong overlap of Ubx and Polycomb complex binding sites in entire embryos. (A-D) UCSC Genome Browser screenshots of Ubx and mock ChIP-seq tracks at known PRE/TRE elements (purple shading) at BX-C, ANTP-C, *en*, *ph-p* and *ph-d*. The coordinates are from [60]. Pho track is from [68]. (E) The box plots show the Pc and Pho ChIP-chip signal (ChIP/input ratio [log2]) [67] at the Ubx peak summits and at control regions. The left panel represents all regions, the middle panel positions that overlap HOT regions and the right panel those that do not overlap HOT regions. NS–not significant; Wicoxon test: **P<10^−20^; equivalent plots for other TFs are in S3 Fig. (F) The plots show the percentage of TFs binding sites that have Pc or Pho signal [67] greater than a given threshold value (X-axis; red line: Ubx, black: control regions and grey: other TFs from [14,24]).

Chromosome 3R also contained two Ubx binding site clusters with 36 and 26 binding sites per 100kb near the *corto* and *taranis* (*tara*) gene loci (Fig 4C). Corto and Tara are Polycomb- and Trithorax-interacting proteins, respectively [55–57] and mutant alleles of both genes were shown to enhance the Polycomb/Thritorax mutant phenotypes and affect Hox gene regulation [57,58]. This is particularly interesting, as we observed multiple Ubx binding sites also in the gene loci of many Polycomb and Trithorax complex members (e.g. *trx*, *osa*, *ash2*, *Pc*, *Pcs*, *ph-p* etc.; Fig 5C). In addition, several of the gene loci bound by Ubx are known direct targets of the Polycomb complex, including *elB-noc* locus [59] and *sbb*/*tango8*, which contain a predicted PRE element [60] (see also below).

To assess the functional relevance of the Ubx binding sites in such highly targeted genomic loci (HTGLs), we evaluated their enhancer activities in transgenic embryos [22] and compared them to the activities of Ubx-bound regions outside HTGLs. Interestingly, VTs overlapping at least one of the Ubx-binding sites within HTGLs were significantly more often active during embryogenesis than those overlapping Ubx binding sites outside HTGLs (64 out of 78 [82%] vs 177 out of 264 [67%], hypergeometric p = 0.0066).

Our observation that Ubx binds to clustered sites within HTGLs around important developmental regulators and that most of these binding sites correspond to active enhancers is interesting. While multiple closely adjacent binding sites of homeodomain proteins within a single enhancer might assist cooperative interactions between TFs and assure stable interaction with the enhancer DNA [61], the HTGLs reported here correspond to clusters of several individual enhancers, presumably similar to locus control regions (LCRs; [62,63]) or super enhancers [64]. The control of developmental regulators via many densely spaced enhancers that are bound by Ubx is also interesting given that Ubx itself is an important developmental regulator that determines segment identity.

### A putative link between Ubx and Polycomb targeting

Hox factors are among the best-characterized targets of the Polycomb and Trithorax complexes, which function to maintain repressive or active transcriptional states, respectively throughout development [65,66]. In *Drosophila*, they have been reported to act through specialized genomic elements, called Polycomb or Trithorax response elements (PRE/TREs; [65]), and BX-C contains several well-studied PREs [65,66].

One of the Ubx peaks with highest ChIP enrichment genome-wide co-localized with a known PRE/TRE (genomic coordinates from [60]) near the non-coding gene *bxd* (Fig 5A). Moreover, other well-characterized PREs in Hox loci and near *ph-d*, *ph-p* and *en* [60] also all contained Ubx peaks (Fig 5A–5D).

Given the small number of *in vivo* validated PRE/TREs, we used genome-wide binding data for Polycomb (Pc) and Pleiohomeotic (Pho) [67] to assess more systematically whether Ubx-bound regions were associated with Polycomb complexes genome-wide. Indeed, the enrichment of Pc and Pho binding was higher at Ubx peaks (score ≥5) than at control regions (Wilcoxon test P<10^−29^) (Fig 5E, left panel). The same was true for Ubx binding sites outside HOT regions (Wilcoxon test P<10^−20^) (Fig 5E, right panel), suggesting that observed association with Pc and Pho was specific to Ubx binding and did not result from general DNA accessibility and protein-DNA association found at HOT regions. Indeed, among 43 different developmental TFs [14,24], Ubx together with Tin, Shn, Kni and Tll had the highest proportion of binding sites overlap with Pc and Pho ChIP signals across a wide range of cut-offs (Fig 5F and S2 Fig). When we directly assessed Pc and Pho ChIP enrichments at binding sites for Ubx and these 43 TFs [14,24], they were higher on average at Ubx binding sites (S3 Fig). The same was true when analyzing independent dataset for Polycomb-associated proteins binding and the Polycomb-associated histone modification H3K27me3 (S2 Fig).

Considering the duality of PRE/TREs, which can switch from activation to repression and our observation that Ubx binding is predictive of active enhancers, we decided to assess the ability of PRE/TREs to enhance expression of a reporter depending on the presence of Ubx. For this we evaluated the activities of VT fragments overlapping 407 PREs (as defined by Pho binding in embryos [68]). Interestingly, 63.4% of Pho-bound VTs (71 out of 112) were active at any stage of embryonic development in comparison to the 46% positive rate for VTs overall (hypergeometric p = 1.4x10^-8^). The percentage of regions acting as enhancers in *Drosophila* embryos increased to 77.5% (31 out of 40) when considering VTs co-bound by Pho and Ubx (score ≥3, summit +/- 250 bp) in contrast to 55.6% (40 out of 72) for VTs bound only by Pho. Such an increase in proportion of positive VTs (63.4% to 77.5%) could happen by chance in less than 1.6% of the cases (hypergeometric p = 0.016).

Moreover, Ubx sites (score ≥3) that overlapped Pho-bound regions [68] were 2.3-fold enriched (p-value<10^−10^) in Pho motifs (GCCATT) [69] unlike Ubx peaks that did not overlap Pho regions (0.8-fold, p-value = 0.001) and even higher than the centers of the broad Pho-bound regions [68] (1.6-fold, p-value = 1.7x10^-7^).

Taken together, these results suggest that Ubx might acts jointly or mutually exclusively with Pc proteins on putative PRE/TRE leading to activation of such genomic regions.

Our ChIP-seq data from entire embryos and different embryonic stages show that Ubx and the Polycomb complex bind to the same genomic regions, suggesting a dynamic interplay between Ubx and Polycomb recruitment. This could occur in parallel or spatially or temporally exclusive domains with different mechanistic implications as we discuss below.

## Discussion

Here we present a *Drosophila melanogaster* strain with a Ubx allele that is tagged at the Ubx C-terminus with a V5 peptide and allowed us to study Ubx binding genome-wide. The tag also contains a biotin-ligase-recognition peptide (BLRP), which should be useful for biochemical approaches, including the biotin/streptavidin-based purification of Ubx containing protein complexes [70,71] and might allow–combined with the targeted expression of biotin ligase (BirA)–to perform tissue-specific ChIP-seq experiments.

We further present a high-quality ChIP-seq dataset that allowed the identification of individual Ubx binding sites genome-wide. These binding sites frequently overlap with active enhancers and Ubx binding is predictive of enhancer activity, especially outside HOT regions. Importantly, Ubx binds extensively to HTGLs, which often overlap the gene loci of developmental regulators and genes that are regulated by the Polycomb complex and the majority of these binding sites are functioning as enhancers during embryogenesis.

Our observation that Ubx binds to known PREs/TREs and that Ubx binding sites also show a significant Pc and Pho ChIP signal is suggestive of a model in which Ubx could be upstream of Pc targeting and involved in mediating or antagonizing Pc and Pho recruitment to their genomic binding sites. The data are consistent with two scenarios: Ubx and Pc/Trx binding might occur predominantly in the same cells and Ubx could be involved in recruiting Pc/Trx to their binding sites. Alternatively, Ubx and Pc/Trx might occur predominantly in mutually exclusive spatial domains or at different stages in the developing embryo and Ubx could potentially counteract Pc binding or functionally antagonize Pc activity [72]. Our data suggest that some genomic regions might have a dual role, functioning as both enhancers (i.e. in activating transcription) and PREs/TREs (i.e. in transcriptional memory), potentially depending on cell type and/or developmental stage.

The first hypothesis is consistent with known Polycomb-dependent Ubx repression by high transient levels of Ubx in haltere [73] and the known repression of *bxd* in Ubx-expressing cells, which involved components of the Trx complex [74]. Our finding that Ubx was bound at *bxd* locus suggests that this repression could be direct and mediated by the Hox factor.

On the other hand, Ubx binding has not been observed at the *Abd-A* and *abd-B* loci in haltere [12], a tissue in which *Abd-A* and *abd-B* are repressed by Pc. Similarly, sites that are bound by Ubx in embryos have high levels of Pc and H3K27me3 in S2 cells (S2 Fig) that do not express Ubx [75]. Therefore, while Ubx could be involved transiently during initial steps of Pc recruitment, it does not seem to be required for repression and Pc might even restrict TF access to these loci [12]. Moreover, as Pc is typically associated with repression, the strong enrichment of active enhancers at Ubx binding sites suggests that Ubx could counteract Pc, potentially through enhancer activation. In other cells, Pc would then bind to and silence the same regions thereby counteracting Ubx function, leading for example to the high levels of H3K27me3 observed in ChIP experiments from entire embryos.

The prediction that Ubx might be involved in specifying or counteracting the recruitment of Polycomb to specific genomic loci is attractive as it links Hox genes, which are involved in the definition of segment identity with Polycomb, which has been implicated in the maintenance of transcriptional regulatory states throughout development. While we find that several TFs co-localize with Pc/Pho binding sites in ChIP from entire embryos (e.g Tin, Shn, Kni and Tll), Ubx had the most prominent effect. Given the attractiveness and potential importance of this link between Hox genes and Polycomb, we would like to share this observation with the broader scientific community.

## Materials and Methods

### Drosophila stocks

Drosophila flies were kept at 25°C on standard food. w^1118^ strain (denoted as mock) was obtained from the Bloomington Stock center.

### Generation of the donor constructs and homologous recombination

The detailed description of P[acman] vector modifications are in [76]. The genomic coordinates of 5’ homology arm: chr3R 12484497–12490226; 3’ homology arm: chr3R 12478739–12484493. The tag included the V5 epitope (see below; in bold), biotin-ligase-recognition-peptide (BLRP) (see below; in italic) separated by PreScission cleavage site (see below; underscored). The DNA sequence of the tag: GCGGCGGCG**GCAAGCCCATCCCCAACC**CCCTGCTGGGCCTGGATAGCACCCTGGAGGTGCTGTTCCAGGGCCCCGAGAACCTGTACTTCCAGGGC*ATGGCCAGCAGCCTGCGCCAGATCCTGGATAGCCAGAAGATGGAGTGGCGCAGCAACGCCGGCGGCAGC*tgaGGTACC. The selection cassette consisted of mini-white gene under hsp70 promoter and GRM enhancer and 2 flanking LoxP sites. The construct was injected into ZH-attP-51D fly strain with the landing site on chr2R [77]. Genetic crosses were done as described in [19]. Positive candidates were confirmed by Southern blot: the restriction enzyme used: XhoI (NEB); 3’ end probe: chr3R 12477423–12478376). The selection cassette removal was done by Cre-mediated recombination [19].

### Embryo collection and ChIP-seq

The Ubx-tagged and w^1118^ flies were kept in large populational cages at 25°C. Embryos were collected for 16 hrs (overnight), dechorionated and frozen. Approximately 1g of frozen embryos was fixed and processed as described [78]. Nuclei were sonicated in 1.5 ml of nuclear lysis buffer [79] with the Tip sonicator (Omni Sonic Ruptor 250 Watt Ultrasonic Homogenizer) for 7 cycles (1 min on [Duty cycle 30%, Output 20%], 1 min off). The average size of sheared fragments was approximately 500 bp. 500 μl of sonicated chromatin was incubated with 25 μl of blocked anti-V5 agarose affinity gel (Sigma, A7345-1ML) and 500 μl of RIPA buffer [79] for 2 hrs at 4°C. The beads were washed as described [79]. A total of 3 ng of material was used for library generation.

### In vivo enhancer activity analysis

All enhancer activity assays are based on transcriptional reporter assays in transgenic embryos available from the Vienna Tile (VT) resource [22] at www.enhancers.starklab.org.

### Deep-sequencing

Sequencing was performed at the CSF NGS Unit (www.csf.ac.at) on an Illumina HiSeq2000 machine. We processed data as single-end sequencing data, compared two independent biological replicates and merged them for the subsequent analyses.

### Reads processing and peak calling

We obtained unique fragments by mapping reads to dm3 genome using bowtie [80], allowing maximum three mismatches. Significantly enriched regions (peaks) were identified using peakzilla [20] with default settings. As a cut-off parameter we used a peak score that takes into account the enrichment and distribution of reads in a peak region [20].

### Data analysis

FDR for ChIP-seq was calculated as (number of peaks in control [WT] ChIP-seq sample/number of peaks in V5 ChIP-seq sample)*100%. We used 1425 negative regions for the analysis that had the same genomic distribution as Ubx peaks (score ≥5). *De novo* motif discovery was done with DREME (-e 0.01) with scrambled or negative regions as background sequences [81]. Motifs enrichments were calculated as in [82]. For analysis in Fig 5E and 5F and S3 Fig we used windowed enrichment ratios (log2Ratios) from studies cited in the text. Either signal at summit positions (Fig 5E) or median signal at the non-HOT peak regions was calculated.

### Data dissemination

The NCBI GEO accession number for the deep sequencing and processed data is GSE64284. All data including tracks in the UCSC genome browser are also available at www.straklab.org.

## Supporting Information

S1 Fig *De novo* motif analysis and clustered Ubx binding sites at the loci of important developmental genes. (A) *De novo* motif recovered in Ubx-bound regions. (B) The number of Ubx peaks per 100 kb on chromosomes 2R, 3L, X and 4 (as in main Fig 4C and 4D). Each 100 kb window starts at a Ubx peak, covering all possible windows that contain at least one Ubx peak. Representative genes for windows containing ≥25 Ubx peaks are indicated.
(EPS)

S2 Fig Association of Polycomb complex with binding sites for developmental transcription factors (TFs). The plots show the percentage of TF binding sites for which the ChIP signal for the indicated Polycomb group protein or the Polycomb-associated histone modification H3K27me3 [68,75,84] is greater than a given threshold value (X-axis; as in main Fig 5F).
(EPS)

S3 Fig Strong association of Pc and Pho with Ubx binding sites. The boxplots show the distributions of Pc and Pho normalized ChIP signal (ChIP/input ratio [log2]) at the binding sites of the indicated transcriptional factors (as in Fig 5E). The binding sites are from [14] and [24] and are restricted to TFs that had Pc and Pho signals significantly higher than control regions. NS–not significant; Wilcoxon test: *P<0.001, **P<10^−5^; #: TF binding sites from [24].
(EPS)
